# The effect of bone cement distribution on the outcome of percutaneous Vertebroplasty: a case cohort study

**DOI:** 10.1186/s12891-020-03568-9

**Published:** 2020-08-13

**Authors:** Lei Tan, Bingtao Wen, Zhaoqing Guo, Zhongqiang Chen

**Affiliations:** 1 Department of Orthopaedics, Peking University International Hospital, Life Park Road No.1 Life Science Park of Zhong Guancun, Changping District, Beijing, 102206 China; 2 Department of Orthopaedics, Peking University Third Hospital, 49 North Garden Road, Haidian District, Beijing, 100191 China

**Keywords:** Osteoporotic vertebral compression fracture, OVCF, Percutaneous vertebroplasty, PVP, Bone cement distribution, Vertebral body height, Vertebral body recompression

## Abstract

**Background:**

To analyze the effect of different types of bone cement distribution after percutaneous vertebroplasty (PVP) in patients with osteoporotic vertebral compression fracture (OVCF).

**Methods:**

One hundred thirty seven patients with single level OVCF who underwent PVP were retrospectively analyzed. The patients were divided into two groups according to bone cement distribution. Group A: bone cement contacted both upper and lower endplates; Group B: bone cement missed at least one endplate. Group B was divided into 3 subgroups. Group B1: bone cement only contacted the upper endplates; Group B2: bone cement only contacted the lower endplates; Group B3: bone cement only located in the middle of vertebral body. The visual analogue scale (VAS) score at 24 h post operation and last follow-up, anterior vertebral height restoration ratio (AVHRR), anterior vertebral height loss ratio (AVHLR), local kyphotic angle change and vertebral body recompression rate were compared.

**Results:**

24 h post operation, the pain of all groups were significantly improved. The average follow-up time was 15.3 ± 6.3 (6–24) months. At last follow-up, the VAS score of group A was lower than that of group B. There were 14 cases (10.2%) of adjacent vertebral fracture, 5 cases (8.6%) in group A and 9 cases (11.4%) in group B. There were 9 cases (6.6%) of cement leakage, 4 cases (6.9%) in group A and 5 cases (6.3%) in group B. At last follow-up, there were 16 cases (11.7%) of vertebral body recompression, including 3 cases (5.2%) in group A and 13 cases (16.5%) in group B. There was no significant difference in AVHRR between two groups. Local kyphotic angle change was significant larger in group B. At last follow-up, AVHLR in group B was higher than that in group A. Analysis in subgroup B revealed no significant difference in VAS score, local kyphotic angle change, vertebral recompression rate, AVHRR or AVHLR.

**Conclusions:**

If the bone cement fully contacted both the upper and lower endplates, it can better restore the strength of the vertebral body and maintain the height of the vertebral body, reduce the risk of the vertebral body recompression and long-term pain.

## Background

With the aging of the population, osteoporotic vertebral compression fracture (OVCF) is becoming more common. Several literatures have confirmed that percutaneous vertebroplasty (PVP) is an effective method for the treatment of such fractures, which can effectively relieve pain, maintain the strength of the vertebral body, and avoid long-term complications from bedridden [1, 2]. However, there are complications such as refracture, loss of vertebral body height and increase of local kyphosis angle, which may be affected by the distribution of bone cement in vertebral body during the first operation. The purpose of this study was to analyze the effect of different types of bone cement distribution on pain relief, vertebral height maintenance, and the rate of vertebral recompression.

## Methods

### General data

Patients with OVCF who underwent single level PVP operation in our institute from June 2016 to June 2019 were analyzed retrospectively. Inclusion criteria: (1) patients with lower back pain as the main manifestation, not accompanied by lower extremity radiation pain, numbness, weakness or other nerve compression symptoms; (2) T-score < − 2.5 in bone mineral density (BMD) examination of lumbar spine, to confirm the osteoporosis; (3) high signal changes in the vertebral body on fat suppression MRI or bone scan examination showed active bone metabolism, to confirm the acute fracture. Exclusion criteria: (1) pathological fracture caused by tumor or infection; (2) patients with severe systemic diseases, unable to tolerate surgery; (3) patients with incomplete data or missing visit. One hundred fifty two patients were initially identified. We enrolled 137 patients and 15 were lost to follow. There were 26 males and 111 females, with an average age of 69 ± 7.0 years old. The average follow-up time was 15.3 ± 6.3 (6–24) months.

### Surgical method

The patient was placed in the prone position and local anesthesia was performed with 1% lidocaine. Under the guidance of C-arm fluoroscopy, the puncture needles were placed through bilateral pedicle paths. The end of the puncture needle was located at 1/3 anterior-mid of the vertebral body on lateral film, and between the inner edge of the ipsilateral pedicle and the midline of the vertebral body on the anteroposterior film. Using a hydraulic injection device, the high viscosity cement was injected slowly under fluoroscopy until the cement was close to the posterior wall of the vertebral body which the leakage was possible. According to medical protocol of our institution, all patients who underwent vertebroplasty would take calcium (100 mg per day), calcitriol (0.5μg per day) and alendronate sodium (70 mg per week) after surgery. The patients were reminded to take medicine on time on regular visit.

### Grouping method

**Fig. 1:**
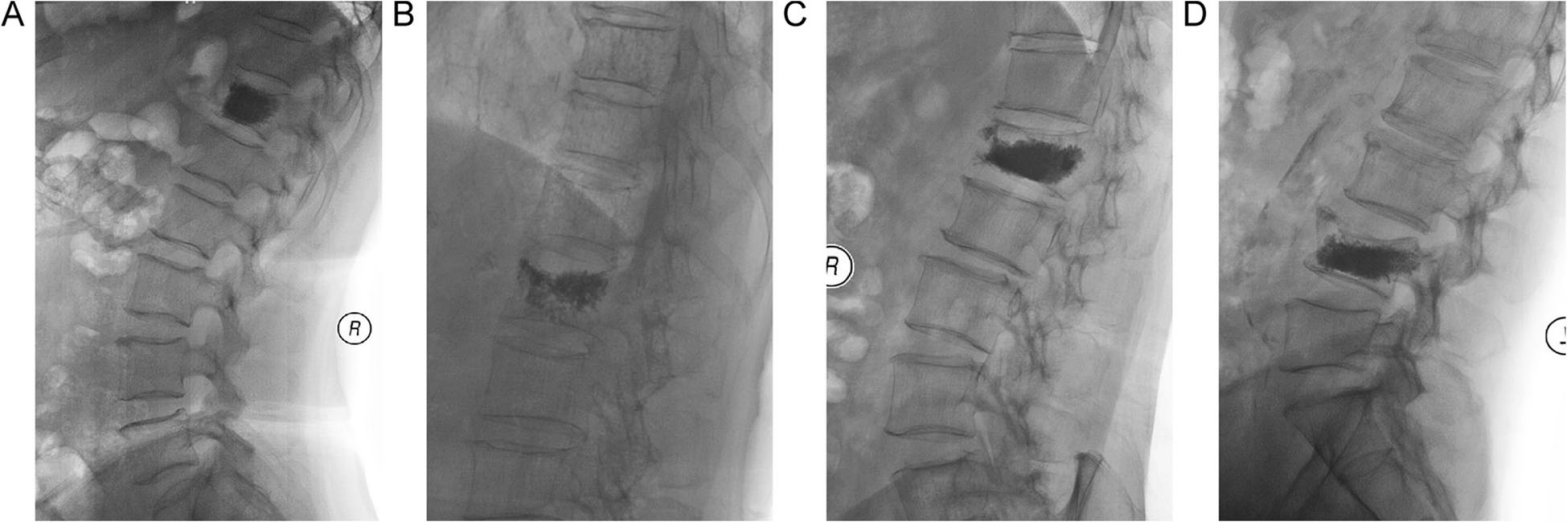
Different types of bone cement distribution. **a.** Bone cement is in contact with the upper and lower endplates. **b.** Bone cement is only in contact with the upper endplate. **c.** Bone cement is only in contact with the lower endplate. **d.** Bone cement is not in contact with the endplate

Radiographs were taken 24 h post operation and patients were divided into two groups according to the distribution of bone cement. Group A: bone cement contacted both upper and lower endplates. Group B: bone cement missed at least one endplate. Group B was divided into 3 subgroups. Group B1: bone cement only contacted the upper endplates; Group B2: bone cement only contacted the lower endplates; Group B3: bone cement only located in the middle of vertebral body. Figure 1 showed illustrations of cement distribution.

### Evaluation method

Age, gender, body mass index (BMI), BMD, fracture segment, vertebral compression degree (mild < 25%, moderate 26–40%, severe > 40%), bone cement volume, adjacent vertebral fracture and bone cement leakage were documented. The visual analog scale (VAS) score pre-operation, 24 h post operation and at the last follow-up were analyzed. Radiography measurement indexes include the anterior vertebral height ratio (AVHR), which was defined as the height of the anterior wall of the compressed vertebral body / (the height of the anterior wall of the upper vertebral body + the height of the anterior wall of the lower vertebral body) × 2. The anterior vertebral height recovery ratio (AVHRR) was defined as postoperative AVHR - preoperative AVHR. The anterior vertebral height loss ratio (AVHLR) was defined as postoperative AVHR – last follow-up AVHR. On the last follow-up, a recompression was confirmed when the height of the anterior wall of the vertebral body deceased more than 1 mm compared with the post operation, or the Cobb angle of the upper and lower endplates increased more than 10°. Local kyphotic angle change was defined as last follow-up Cobb angle of upper and lower endplates – postoperative Cobb angle of upper and lower endplates.

### Statistical method

SPSS 19.0 software (SPSS Inc., USA) was used to analyze the data. The continuous variable was expressed as mean ± standard deviation, and independent sample t-test or variance analysis (ANOVA) was used. Chi square test was adapted to analyze the categorical variable. Significant differences were defined as *p* < 0.05.

## Results

There was no significant difference in age, gender, BMI, BMD, fracture segment, fracture compression degree, bone cement volume and follow-up time between groups (as shown in Table 1).

**Table 1:** Basic information of patients

	Group A (*n* = 58)	Group B1 (*n* = 30)	Group B2 (*n* = 37)	Group B3 (*n* = 12)	*p* value
Age	68.9 ± 8.5	69.8 ± 6.1	69.7 ± 8.7	74.0 ± 6.2	0.248
Gender					0.188
Male	15	2	7	2	
Female	43	28	30	10	
BMI(kg/m^2^)	23.5 ± 4.3	21.6 ± 4.7	22.9 ± 5.2	21.6 ± 2.9	0.253
Bone mineral density (T score)	−3.2 ± 0.4	−3.3 ± 0.3	−3.3 ± 0.3	−3.4 ± 0.3	0.325
Fracture segment					0.585
Thoracic (T1–10)	9	6	4	3	
Thoracolumbar (T11-L2)	41	22	28	5	
Lumbar (L3–5)	8	3	6	3	
Vertebral compression degree					0.820
Mild (< 25%)	25	10	14	3	
Moderate (26 ~ 40%)	16	10	12	6	
Severe (> 40%)	17	10	11	3	
Bone cement volume (mL)	6.0 ± 1.3	6.1 ± 1.0	5.6 ± 0.9	5.7 ± 1.1	0.247
Follow up time (months)	16.2 ± 6.5	14.7 ± 6.1	14.0 ± 5.9	16.0 ± 7.2	0.374

The pain was significantly relieved and there was no statistical difference in pre-operation or 24 h post-operation VAS score between group A and B. At the last follow-up, the VAS score of group A was statistically lower than that of group B. There were 14 cases (10.2%) of adjacent vertebral fracture, 5 cases (8.6%) in group A and 9 cases (11.4%) in group B. There were 9 cases (6.6%) of bone cement leakage, 4 cases (6.9%) in group A and 5 cases (6.3%) in group B. There was no statistical difference between two groups. At the last follow-up, there were 16 cases (11.7%) of vertebral recompression, including 3 cases (5.2%) in group A and 13 cases (16.5%) in group B. There was statistical difference between the two groups. Local kyphotic angle change was significant larger in group B. There was no significant difference in AVHRR between the two groups. At the last follow-up, AVHLR in group B was significantly higher than that in group A (Table 2).

**Table 2:** Analysis of outcome between different groups

	Group A	Group B	*p* value
VAS (pre-op)	6.1 ± 1.7	5.7 ± 1.5	0.157
VAS (24 h post-op)	2.0 ± 1.2	1.9 ± 1.1	0.641
VAS (last follow-up)	1.4 ± 1.1	2.0 ± 1.2	0.002*
Adjacent vertebral fracture (%)	8.6% (5/58)	11.4% (9/79)	0.597
Bone cement leakage (%)	6.9% (4/58)	6.3% (5/79)	0.895
Local kyphotic angle change (°)	3.4 ± 3.9	4.9 ± 4.1	0.029*
Recompression (%)	5.2% (3/58)	16.5% (13/79)	0.042*
AVHRR (%)	6.6 ± 4.0	5.8 ± 3.9	0.241
AVHLR (%)	4.0 ± 2.6	6.8 ± 3.8	< 0.001*

*there was statistical difference when *p* < 0.05

**Fig. 2:**
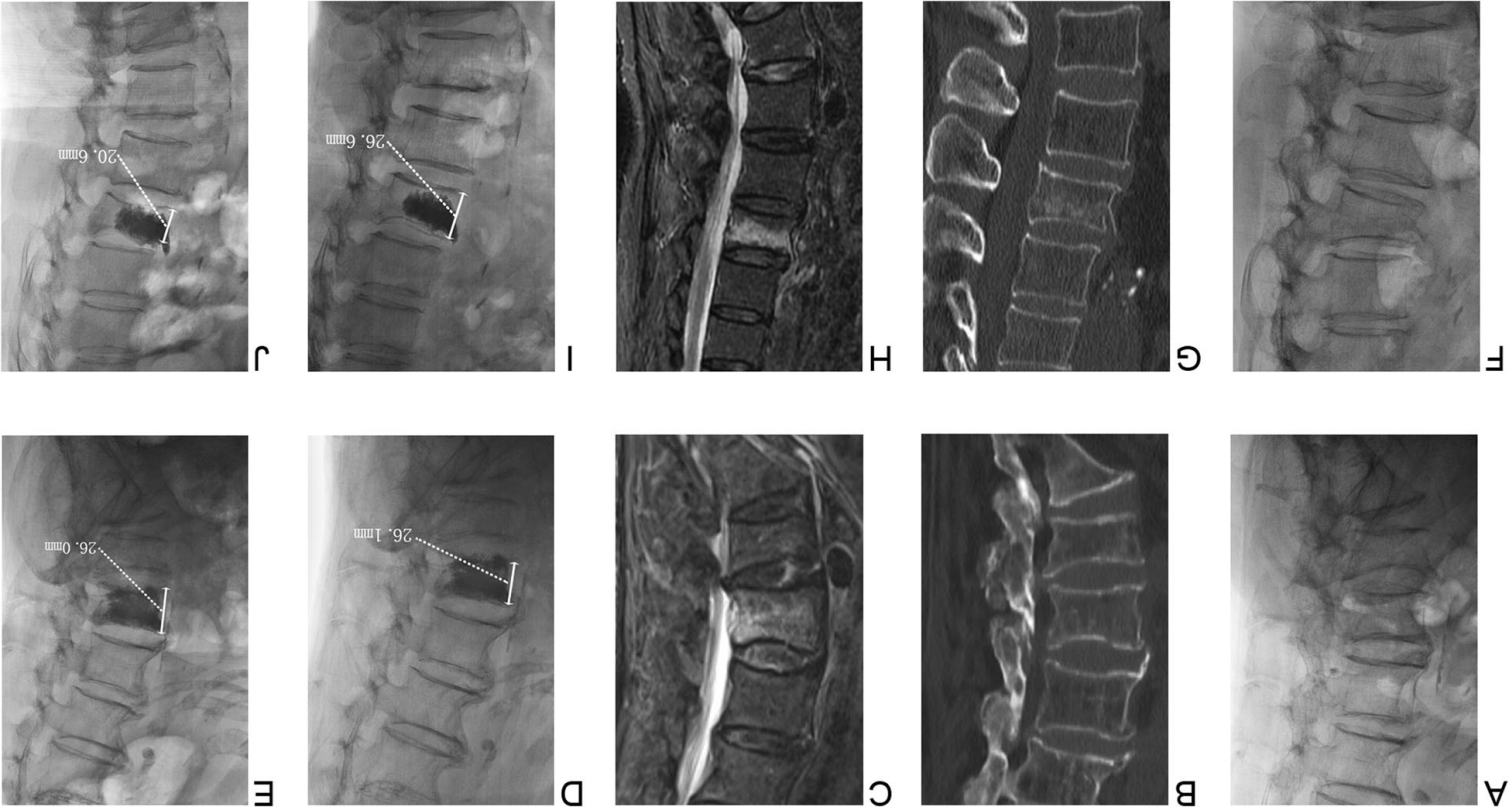
Typical cases. A 71 years old female patient, preoperative x-ray (**a**), CT (**b**), MRI (**c**) showed acute OVCF of L4. X-ray (**d**) at 24 h post operation showed that the cement was in close contact with the upper and lower endplates, and X-ray (**e**) at 12 months post operation showed that the vertebral height was maintained well. A 62 years old female patient, preoperative x-ray (**f**), CT (**g**), MRI (**h**) showed acute OVCF of L2. The X-ray (**i**) at 24 h post operation showed that the cement did not contact the lower endplate. The X-ray (**j**) at 6 months post operation showed that the height of the vertebral body was lost and the vertebral body was recompressed

Analysis in subgroup B revealed no significant difference in VAS score, adjacent vertebral fracture rate, bone cement leakage rate, local kyphotic angle change, vertebral recompression rate, AVHRR or AVHLR (Table 3). Typical cases were shown in Fig. 2.

**Table 3:** Analysis of outcome between sub-Group B

	Group B1	Group B2	Group B3	*p* value
VAS (pre-op)	5.8 ± 1.5	5.8 ± 1.4	5.3 ± 1.8	0.542
VAS (24 h post-op)	2.1 ± 1.2	1.8 ± 1.1	1.6 ± 1.2	0.457
VAS (last follow-up)	1.7 ± 1.3	2.4 ± 1.3	1.8 ± 1.6	0.072
Adjacent vertebral fracture (%)	16.7% (5/30)	8.1% (3/37)	8.3% (1/12)	0.513
Bone cement leakage (%)	6.7% (2/30)	8.2% (3/37)	0%(0/12)	0.602
Local kyphotic angle change (°)	4.7 ± 3.5	4.8 ± 4.4	5.7 ± 5.1	0.777
Recompression (%)	13.3% (4/30)	16.2% (6/37)	25%(3/12)	0.653
AVHRR (%)	5.4 ± 3.8	6.6 ± 3.7	4.7 ± 4.2	0.248
AVHLR (%)	7.6 ± 4.0	6.0 ± 3.9	7.1 ± 2.5	0.213

## Discussion

OVCF is commonly seen in elderly patients. In a multicenter prospective study involving 2451 elderly women, 32% of the patients had at least one vertebral compression fracture [3], which may occur with or without slight trauma. The bone cement can restore the strength of the fractured vertebral body and produce thermal necrosis effect on the pain nerves in the vertebral body. It is an effective way to treat OVCF [4, 5] and affected by many factors such as the patient’s BMD, the volume and distribution of bone cement [6]. Biomechanical tests have shown that restoration of strength and stiffness required vertebral body cement fills of 16.2 and 29.8%, respectively [7]. It is not only the volume of bone cement, but also the distribution of bone cement in the vertebral body has an important influence on the effect of operation and the long-term maintenance of the vertebral body height.

Some literatures classified the bone cement distribution according to its diffusion on anteroposterior X-ray film, and then their influence on the long-term outcome was studied [8]. However, this classification method was mostly applicable for unilateral puncture cases, because bilateral puncture injection of bone cement can often achieve a uniform distribution on both sides of the vertebral body, effectively avoiding the uneven stress caused by asymmetric distribution on the coronal plane. Many previous literatures have also confirmed that bilateral puncture does not significantly increase the risk of complications such as cement leakage and nerve injury as long as the puncture route is strictly followed [9, 10]. Therefore, studying the distribution of bone cement in the sagittal plane on lateral radiographs may be more meaningful for the analysis of surgical efficacy.

Our study found that if bone cement can fully contact with the upper and lower endplates, it can better maintain the height of the vertebral body and reduce the risk of vertebral recompression. In our study, the surgical vertebral recompression rate in Group A was 5.2%, which was significantly lower than that in group B (16.5%). Previous literatures reported a surgical vertebral recompression rate of 3.2% ~ 27.6% with different criteria of recompression and follow-up time [11–13]. This kind of surgical vertebral recompression is multifactorial, often without a clear traumatic event, may be related to the degree of osteoporosis, daily activities and the distribution of bone cement [14]. Insufficient filling of bone cement is an important cause of recompression, especially the uneven distribution in the sagittal plane [15]. If the cement is in full contact with the upper and lower endplates, it will fill the whole vertebral body and play a full role of “bonding” for the cancellous bone and the endplate, which can better restore the strength of the vertebral body [16]. When the cement only touches the upper or lower endplates, vertebral strength only increases about 2 times. If the cement touches the upper and lower endplates at the same time, it will prompt 8–12 times and significantly improve the stress transmission [17]. In Kim’s study, 46.7% (7/15) of the patients have a recompression at an average of 3.4 months post operation if the cement had no contact with the endplate [18]. Our finding is consistent with Liang, after an average follow-up of 29.6 months, 8.16% or 37.4% of the patients will have surgical vertebral recompression if the cement is in full contact with the upper and lower endplates or not in their study [19].

Analysis in subgroup B revealed the recompression rate was higher in group B3 when bone cement contacted none of the endplate, but there is no statistical difference, which may due to the relatively small sample size. The upper and lower endplates are equally important. The lack of contact between the bone cement and any endplate will result in an unfilled vulnerable area, where recompression usually happened.

In our study, regardless of the type of cement distribution, it has obvious benefits for short term pain relief and recovery of vertebral height. And this is consistent with previous clinical experience [20]. However, in the long-term follow up, the degree of pain in group A were significantly lower than group B. Ye revealed insufficient filling of bone cement was associated with chronic lower back pain [21]. Recompression may lead to changes in spinal balance, local kyphosis, and consequently chronic pain. Especially when there is a fracture in vertebral endplate, if the cement is not well connected with the endplate, it will provide insufficient support and lead to the continuous compression of the fracture vertebral body, which is the reason for the persistence of postoperative pain [22]. He found that the long-term effect of H-type distribution of bone cement is better than O-type distribution, which is related to the closer contact between bone cement, endplates and cancellous bone in H-type distribution [23]. Our study and previous literatures show that if the bone cement is evenly distributed and closely contacted with the upper and lower endplates, it can better maintain the strength and height of the vertebral body, reduce recompression risk and eventually improve the patients’ chronic back pain.

It is not an ideal way to obtain a wide distribution of bone cement by increasing excessive cement volume. Because laboratory based biomechanical study found that the stiffness of the injured vertebral body can be restored when the volume of bone cement reaches approximately 15% of the vertebral body. If the volume of bone cement injected is increased beyond this value, there is no significant benefit, and it may cause asymmetric distribution of bone cement and excessive rigidity of the vertebral body [24]. The clinical usage of cement volume can be excessive than 15%, nonetheless, few significant benefits have been shown when the volume reaches to beyond 24% of the vertebral body, at which point that can already effectively relieve the pain [25, 26]. In our study, no significantly difference of bone cement volume was found between groups. Additionally, an increase in the cement volume may increase the risk of cement leakage [27–29]. Bone cement volume is only weakly related to the effect of the operation [30] and it is not advisable to increase the volume of bone cement excessively. Compared with percutaneous kyphoplasty (PKP), PVP may achieve better cement distribution. Loss of vertebral height was more likely after PKP than PVP [31, 32]. Because balloons squeeze cancellous bone around during expansion in PKP, creating a “cavity”. Cement tends to distribute in this low-pressure cavity without infiltrating into the surrounding bone, making it difficult for cancellous bone to bond tightly, and this mass-like cement distribution has also been proved to be risk factor of recompression [33].

In addition, when puncturing bilaterally, the angle of puncture needle can be adjusted so that the two puncture needles point to the upper or lower endplate respectively [34], using high-viscosity cement [35], using hydraulic assistant device to inject bone cement slowly and uniformly may be more beneficial for a better distribution of bone cement [36]. High-viscosity cement has the advantages of fast bonding with bone, long working time window and low polymerization temperature [37]. A meta-analysis found that high-viscosity cement has significant advantages in pain improvement, recovery of cobb angle and cement leakage comparing with low-viscosity cement [38].

There are some limitations in this study, the retrospective study and relatively small sample size may produce some bias. Prospective studies with large numbers of cases are needed to further clarify the relationship between cement distribution and surgical outcome.

## Conclusions

Whether or not the cement is in full contact with the upper and lower endplates, it can have a good immediate analgesic effect. However, if the bone cement fully contacted both the upper and lower endplates, it can better restore the strength of the vertebral body, and then better maintain the height of the vertebral body, reduce the risk of the vertebral body recompression, and its long-term effect is better.

## Data Availability

The datasets used during the current study are available from the corresponding author on reasonable request.

## Abbreviations

PVP: Percutaneous vertebroplasty
OVCF: Osteoporotic vertebral compression fracture
VAS: Visual analogue scale
AVHRR: Anterior vertebral height restoration ratio
AVHLR: Anterior vertebral height loss ratio
BMI: Body mass index
BMD: Bone mineral density
PKP: Percutaneous kyphoplasty
